# Actin-Based Cell Protrusion in a 3D Matrix

**DOI:** 10.1016/j.tcb.2018.06.003

**Published:** 2018-10

**Authors:** Patrick T. Caswell, Tobias Zech

**Affiliations:** 1 Wellcome Trust Centre for Cell-Matrix Research, Faculty of Biology, Medicine and Health, University of Manchester, Manchester Academic Health Science Centre, Manchester, UK; 2 Department of Cellular and Molecular Physiology, Institute of Translational Medicine, University of Liverpool, Liverpool, UK

**Keywords:** migration, invasion, protrusion, lamellipodia, filopodia, actin

## Abstract

Cell migration controls developmental processes (gastrulation and tissue patterning), tissue homeostasis (wound repair and inflammatory responses), and the pathobiology of diseases (cancer metastasis and inflammation). Understanding how cells move in physiologically relevant environments is of major importance, and the molecular machinery behind cell movement has been well studied on 2D substrates, beginning over half a century ago. Studies over the past decade have begun to reveal the mechanisms that control cell motility within 3D microenvironments – some similar to, and some highly divergent from those found in 2D. In this review we focus on migration and invasion of cells powered by actin, including formation of actin-rich protrusions at the leading edge, and the mechanisms that control nuclear movement in cells moving in a 3D matrix.

## Highlights

Cell migration has been well studied in 2D, but how this relates to movement in physiological 3D tissues and matrix is not clear, particularly in vertebrate interstitial matrix.

In 3D matrix cells actin polymerisation directly contributes to the formation of lamellipodia to facilitate migration and invasion (mesenchymal movement), analogous to 2D migration; actomyosin contractility promotes bleb formation to indirectly promote protrusion (amoeboid movement).

Mesenchymal migration can be characterised by polymerisation of actin to form filopodial protrusions, in the absence of lamellipodia.

Translocation of the nucleus is emerging as a critical step due to the constrictive environment of 3D matrices, and the mechanisms that transmit force to the nucleus and allow movement are beginning to be uncovered.

## Cell Migration: On or in Extracellular Matrix?

Cell migration on 2D surfaces, including tissue culture plastic and glass, has been formalised into a cascade of steps that starts with the establishment of polarity and formation of protrusions at the front of cells, with retrograde flow of actin providing traction force for forward movement and completion of the cycle by retraction of the trailing edge [1]. Cell culture has historically been performed in 2D plates as they are more accessible to microscopy and biochemical isolation. *In vivo*, migrating cells can encounter 2D surfaces (e.g., lining of body cavities, as experienced by migrating haemocytes in *Drosophila*). The suitability of 2D plastic/glass surfaces as representative biological models has been questioned in recent years due to their incredibly high rigidity compared to any surface *in vivo* (other than bone), and the simplicity of extracellular matrix (ECM) presentation when compared to complex fibrillar **interstitial matrix** (see Glossary), for example, the connective tissue of vertebrates. In this review, we focus on the functions of actin in cell motility within a **3D matrix**, with particular attention on the migration of cancer cells through an interstitial matrix (a key step in metastasis). Because the unrestricted movement of cells on 2D surfaces has enabled a detailed understanding of the basic machinery that cells use to achieve progressive motion, we first introduce this fundamental machinery and highlight recent advances that might be relevant to future studies in 3D systems. We outline the key mechanisms that underpin different modes of actin-based protrusion in 3D matrices, and where these reflect movement in 2D systems. Finally, we discuss the function of actin polymerisation in coordinating movement of the nucleus, considered the key step in translocation of the cell.

## Understanding Actin in Migration: Lessons from 2D

**Figure 1:**
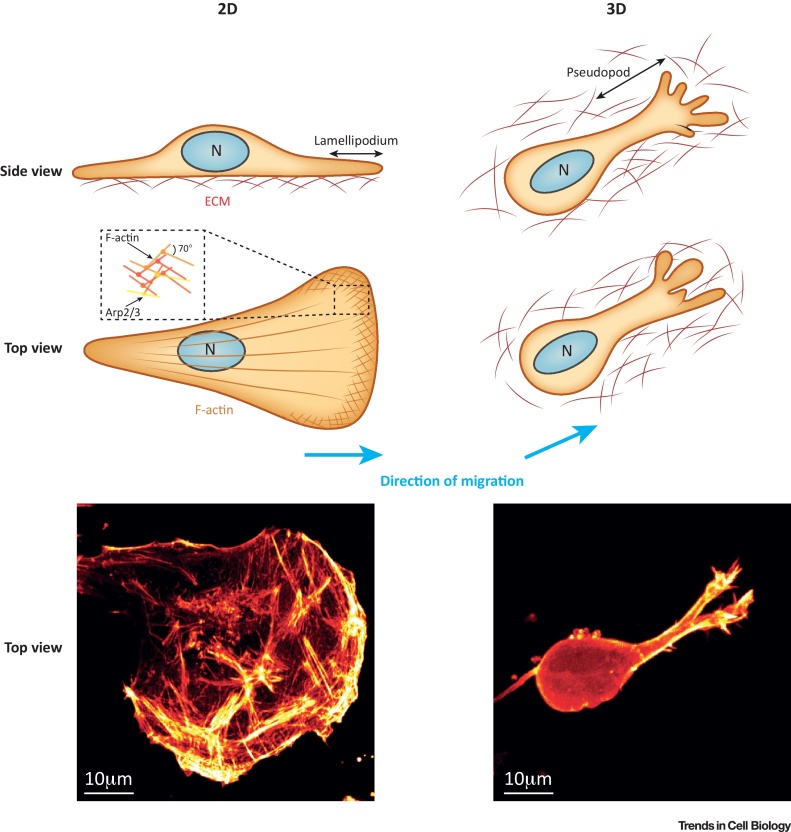
Cell Morphology and Matrix Topology in 2D versus 3D Systems. Cells migrating in 2D and 3D systems encounter different terrains, and adopt morphology suited to these. On flat 2D surfaces, cells encounter extracellular matrix molecules (exogenously added, from serum, and/or secreted by the cell) bound to the planar substrate and engage these through adhesion complexes. This leads to formation of flat lamellipodia via signalling cascades generated by adhesion complexes and other cell surface receptors, which create a dendritic network of actin filaments catalysed by the branching action of the Arp2/3 complex that polymerises actin filaments at a 70° angle from existing filaments [see inset: round shapes represent the Arp2/3 complex, lines F-actin (barbed ends to the right)]. Polymerisation of actin in such networks establishes retrograde F-actin flow and contributes to the generation of traction force. In 3D matrices, such as interstitial extracellular matrices encountered by metastatic cancer cells, cells encounter arrays of fibrillar matrix macromolecules (representative of interstitial matrix, with fibrillar collagen as a key structural component) that act as a barrier to migration, and often extend numerous long processes (known as pseudopods) tipped by actin-based protrusions (including lamellipodia and filopodia) through pores in the matrix. Bottom panels: cancer cells migrating on a 2D surface or within a 3D collagen hydrogel (Lifeact–GFP expressing cells, maximum intensity projections of z stacks captured by spinning disk confocal microscopy; images captured by P. Caswell). Abbreviation: N, nucleus.

The most iconic form of protrusion formed by cells is the large fan-like structures called **lamellipodia**, whose formation is regulated by small GTPases of the **Rho** family and an interconnected network of **WASP**, **Ena/VASP**, and **formin** families of actin regulators 1, 2. **Arp2/3** mediates the assembly of a dendritic F-actin network in lamellipodia (Figure 1), and is activated by members of the WASP family. The WASP family member WAVE can act in a complex with Ena/VASP family proteins, which bind the polymerising barbed end of actin filaments to prevent capping and support optimal actin polymerisation efficiency [2]. Arp2/3-mediated actin polymerisation and actomyosin contractility generate retrograde flow of F-actin, which when engaged by a ‘clutch’ (**focal adhesions)** promotes traction force [3]. Formins can act as direct RhoGTPase effectors to polymerise and/or bundle F-actin from the barbed end [2], and generate actin cables supporting the lamellipod area and force generation 4, 5, 6. Polymerisation and bundling of a subset of linear actin filaments within needle-like protrusions (rather than fan like lamellipodia) forms a class of F actin-based protrusions broadly termed **filopodia**, and numerous pathways can lead to their formation. These include convergent elongation from Arp2/3-generated dendritic actin networks, and direct polymerisation of actin from the barbed ends by formins, with critical supporting roles for Ena/VASP family members and actin-bundling proteins also identified 7, 8. Filopodia can align with focal adhesions, but it is not clear if the filopodial actin structure is force generating/bearing, or if the role is more closely linked to direction sensing. Emerging evidence suggests that a number of subtypes of filopodia exist that could fulfil each of these functions [9].

### Emerging Features of Actin-Based Protrusion in 2D

Recent studies have supported the notion that an as-yet-unexplored level of complexity and coordination exists within actin networks formed in cells migrating on 2D surfaces. The isoforms of the basic building blocks of actomyosin networks were long thought to be randomly incorporated but have been shown to have much more isoform specificity than previously thought. α-, β-, and γ-Actins show distinct distribution in fibroblasts [10] and neurons [11] and are thus likely to support specific functions. The heptameric Arp2/3 actin nucleation complex has intrinsic mechanisms to change its efficiency in actin polymerisation with the utilisation of ARPC1, 3, and 5 isoforms 12, 13, adding further intricacy to the migratory machinery. In addition, non-muscle myosin IIA and IIB, the key motor proteins in the contractile actin cytoskeleton which generates F-actin retrograde flow, are distributed in a potentially self-organising front–rear gradient in polarised migrating cells 14, 15.

Once established, the dynamics of 2D actin-based protrusions are controlled by feedback mechanisms that control the establishment of novel protrusions or the properties of existing ones [16]. Feedback loops can result from signalling networks within conventional Rho GTPase networks [17]. More recently, actin networks have been shown to adjust to mechanical challenges by increasing network density resulting in higher force generation [18] and changes in geometry [19]. Feedback into existing actin structures can be both positive and negative, and more dedicated negative regulators of Arp2/3-mediated actin polymerisation, including Gadkin and Arpin, have been uncovered, which are able to influence protrusion behaviour 20, 21. These feedback mechanisms will be even more significant when superimposed on the restricted environment of confined migration in 3D matrices.

Given the macromolecular arrangements in lamellipodia, one might expect coordinated recruitment of regulatory factors, and recent evidence indicates that the recruitment of such factors can either be driven by diffusion and/or directed recruitment. In support of the latter, microtubule persistence was recently shown to be required for pseudopod maintenance [22]. Microtubule-based transport in turn is influenced by distribution of the membrane tethering exocyst complex [23] and the exocyst complex can influence the recruitment/retention of Arp2/3 [24] and interact with the WAVE and WASH complexes 25, 26.

## Mechanisms of Migration in 3D Microenvironments

**Figure 2:**
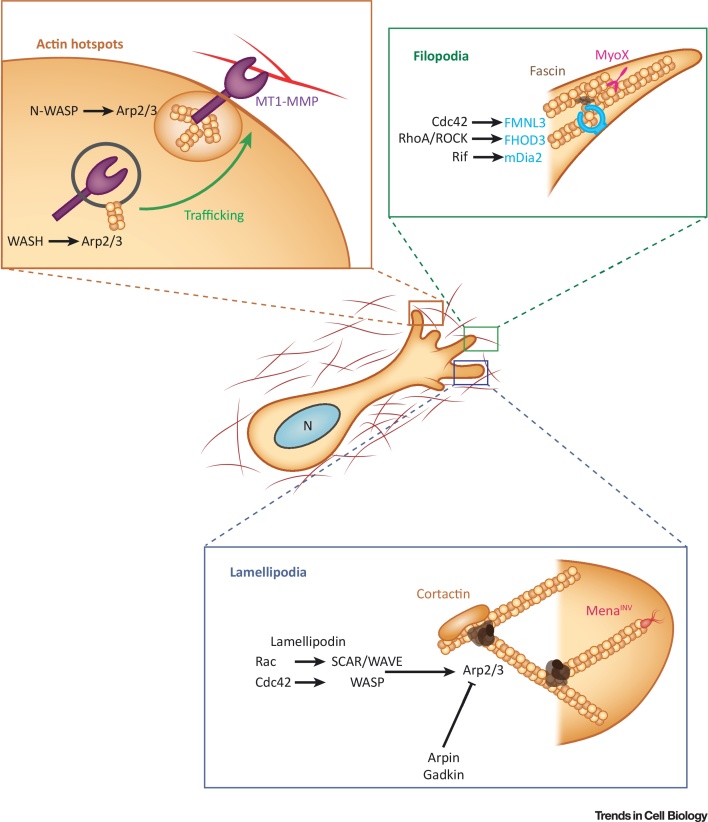
Actin-Based Protrusion in 3D Migration. Mesenchymal cells in 3D matrices use actin to protrude by generating lamellipodia (smaller in scale to those seen in 2D), filopodia, and by anchoring matrix proteases at the cell surface within ‘actin hotspots’. The signalling network upstream of lamellipodia (blue box) is analogous to that in 2D, whereby Rac (or Cdc42) can activate WASP family members to promote Arp2/3 (brown complex) activity and formation of a branched actin network. Cortactin (orange) can play a key role in coordinating Arp2/3 nucleated actin polymerisation, and Mena^INV^ supports filament elongation to promote 3D migration. Filopodia (green box) can be generated via alternate pathways, including through formins (blue) that dimerise to polymerise actin from the barbed end. Cdc42 mediated activation of FMNL3, RhoA/ROCK-mediated phosphorylation and activation of FHOD3, and Rif-mediated activation of mDia2 have each been shown to promote migration in 3D contexts. Other factors [e.g., fascin (brown), MyoX (pink)] play key roles in bundling actin filaments within filopodia. The WASH complex promotes actin-dependent trafficking (green arrow) of MT1-MMP to N-WASP-Arp2/3 nucleated ‘actin hotspots’ (orange box and circle) within pseudopods, where MT1-MMP can degrade matrix to promote invasion. Abbreviations: FMNL3, formin-like 3; MyoX, myosin X.

On **2D substrates**, cells encounter, adhere to, and generate force against a single surface. In 3D microenvironments the terrain, in terms of the topology, rigidity, and uniformity encountered, is vastly different (Figure 1). **Basement membranes** form thin sheet like structures that provide anchorage for epithelial and endothelial cells (among others) and separate tissues/organs from underlying **interstitial matrix**, a complex 3D structure dominated by fibrillar collagens that contains pores of varying sizes that can allow egress/entry of migrating cells. Hence, it is perhaps unsurprising that cells can adopt a variety of migratory modes in a 3D matrix, which describe the morphological appearance and/or mechanism of protrusion/propulsion [27]. Moreover, cells within 3D-ECMs show a remarkable degree of plasticity and are able to switch migration mode depending on both intrinsic and extrinsic factors [27]. The ability of cells to move in collective sheets or strands adds further complexity to migratory behaviours [28]. Here we focus on single cell migration and mechanisms of actin-based protrusion (Figure 2); however, it is likely that the mechanisms of protrusion at the leading edge are shared by leader cells in collectively migrating groups of cells.

### Generating Protrusive Force through Hydrostatic Pressure

Cells can move in 3D without initial polymerisation of actin at the leading edge to generate protrusions. Membrane **blebbing** as a means of protrusion in motile cells in 3D ECMs 29, 30 is most likely related to migratory strategies used by leukocytes, which can move independently of conventional adhesion mechanisms [31]. Actomyosin-based contractility toward the rear of the cell generates hydrostatic pressure and flow of cytoplasm to form spherical membrane expansions (blebs), a consistent feature of **amoeboid** migration that facilitates forward movement 32, 33. Such amoeboid cells are generally less dependent on cell–matrix adhesion and protease activity, and importantly, many cancer cells show remarkable plasticity, switching between amoeboid and mesenchymal motility dependent on cell intrinsic and extrinsic factors 27, 34, 35. Stable bleb formation has been shown to drive motility of physically confined cells both *in vitro* and *in vivo*, and stable blebs and hydrostatic pressure are maintained by rearward cortical actin flow 36, 37. Cortical actomyosin contractility and nuclear pistoning in fibroblasts and cancer cells can also drive formation of blunt protrusions, termed lobopodia 38, 39, 40. These modes of motility in 3D-ECMs and *in vivo* are not defined by F-actin-based protrusions, rather by actomyosin contractility, and have been reviewed expertly elsewhere 34, 41.

### Lamellipodium-Based Protrusion in 3D-ECMs

Actin polymerisation is key to migration in 2D, and the mechanisms identified in such systems were long thought to parallel processes which occur in **mesenchymal** cells moving in more complex 3D environments such as those found *in vivo,* including cancer cells (post **epithelial–mesenchymal transition**) and fibroblasts (during wound healing; Figure 1). In support of this, use of photoactivatable Rac in zebrafish neutrophils to induce WAVE/Arp2/3 activity demonstrated that acute induction of Rac activity can promote leading edge protrusion *in vivo* [42]. Regulators of Arp2/3 and lamellipodia formation have also been implicated in cancer metastasis in human patients and mouse models; for example, overexpression of WASP family members is associated with poor outcomes 43, 44, 45, 46, 47, 48, and decreased expression of the Arp2/3 negative regulator Arpin is associated with poor prognosis in breast cancer [45]. Furthermore, a splice variant of the actin regulator Mena, Mena^INV^, is associated with breast cancer metastasis 49, 50 and plays a clear role in **haptotaxis** in 3D matrices 51, 52. In addition, **lamellipodin** is implicated in breast cancer metastasis, and controls actin reorganisation and lamellipodia formation by interacting with WAVE and Ena/VASP family members to control protrusion *in vivo* and mediate tumour dissemination [53]. N-WASP can compensate for loss of WAVE in cancer cells, and in fact promote invasive migration in 3D through Arp2/3 complex activation [54]. While it is clear that regulators of lamellipodial cell migration are important in cell motility in 3D environments, these regulators also control actin dynamics in other contexts, including endocytosis and **invadopodia** formation 53, 54, 55, 56. Hence, in many cases the direct contribution to leading edge actin reorganisation is not known.

Rac activity is clearly implicated in the migration of mesenchymal cancer cells in 3D and *in vivo* 35, 57. Rac1 knockout melanoblasts show defects in extension of pseudopodial protrusions and cell motility, which contribute to aberrant melanoblast patterning and pigmentation in mice, but long-term *in vivo* migration experiments also reveal the requirement for Rac1 in cell cycle progression, complicating simple interpretation [58]. Inhibition of actin polymerisation or Arp2/3 prevents wild-type melanoblast motility in dermal explants [58], which suggests a direct link between Rac-Arp2/3-driven actin polymerisation and melanoblast migration *in vivo*. Lamellipodin can act as a platform to deliver active Rac to Arp2/3, activating the SCAR/WAVE complex at the leading edge of migrating cells, and is also required for melanoblast motility and correct pigmentation in mice [59]. The lamellipodin–SCAR/WAVE interaction is crucial for neural crest cell migration in *Xenopus* embryos [59], suggesting that lamellipodin indeed controls the Arp2/3-mediated generation of dendritic actin networks to control motility *in vivo*. Cdc42 also plays a key role in melanoblast migration in mouse skin, and while Cdc42 null melanoblasts are able to elongate and adopt a mesenchymal morphology, they are unable to efficiently move [60]. This is due to defects in formin and Arp2/3-mediated actin assembly, adhesion complex dynamics, and active myosin localisation, reflecting the broad effector pathways downstream of this Rho GTPase.

In 3D collagen gels, cancer cells generate pseudopodial protrusions that are reliant on Arp2/3, N-WASP, WAVE1, cortactin, and Cdc42, although broad lamellipodial protrusions were not identifiable in that study [61]. High-resolution, spinning disk confocal imaging within collagen gels has revealed the presence of cell–matrix adhesion complexes within small lamellipodia-like protrusions of fibroblasts [62], and small lamellipodial protrusions are readily detectable, and retrograde flow of actin is observed, in cancer cells within cell-derived matrices [63].

### Filopodia: Forging the Way

While increased lamellipodial activity has been suggested to promote 3D migration, invasion, and metastasis, there is evidence that lamellipodial regulators (including the Rac activator Tiam-1 and WAVE complex components) are downregulated in metastatic cancer 64, 65, 66, 67, and it is therefore likely that other forms of F-actin-based protrusion can complement or compensate to effect migration in 3D. Filopodia have been reported to serve numerous purposes in migrating cells, including sensing the chemical and physical environment, facilitating cell–cell adhesion in zippering epithelial sheets, and forming protrusions [7]. Filopodia formation has also been implicated in cancer invasion and metastasis; **fascin**, an actin-bundling protein that promotes filopodial formation, is upregulated in numerous metastatic mouse and human tumour contexts 68, 69, 70, 71, 72. Furthermore, **myosin X** expression is induced by expression of gain-of-function mutant p53 to promote metastasis in mouse models of pancreatic cancer, and is linked to poor outcome in breast cancer [73].

The properties of filopodia and the mechanisms that form them have been studied during migration in 3D-ECMs in development and cancer, and have revealed important context-specific differences. In migrating primordial germ cells (PGCs) of the zebrafish embryo, filopodia appear to play a role in sensing chemokines, rather than providing a mechanism for protrusion, ECM adhesion and/or force generation. Filopodia extend toward gradients of CXCL12a, and promote increased pH and Rac activation at the cell front to determine polarised PGC migration in the embryo [74]. However, during sprouting angiogenesis in the zebrafish embryo, formation of filopodia facilitates motility of endothelial tip cells, but is not required for guidance [75]; this suggests that in this context filopodia do not respond directly to chemotactic cues. A more recent study demonstrated that bone morphogenetic protein (BMP) signalling induces expression of ARHGEF9b in endothelial tips cells to activate Cdc42 and generate filopodia via formin like 3 (FMNL3) [76]. FMNL3 has also been implicated in angiogenesis in mammalian systems, suggesting a conserved mechanism. However, while fascin plays a role in F-actin bundling in filopodia in cancer and promotes filopodia formation in endothelial tip cells, its influence on angiogenesis is moderate [77], indicating that this filopodial regulator serves a more redundant role in this cell type.

Filopodia have also been directly observed in invasive and metastatic cancer cells, and their morphology and density may reflect the specific roles they play. A small number of long filopodia-like protrusions (FLPs) are generated around the periphery of mammary carcinoma cells as they enter lung parenchyma and interstitium-like environments [78]. FLPs initiate ECM contact in metastatic breast cancer cells via the combined action of RhoGTPase-formin (Rif-mDia2) and integrin signalling (ILK-Parvin-Pix-Cdc42-PAK-cofilin) axes to increase FLP lifetime, facilitating adhesion formation and proliferative signals via FAK–ERK, promoting tumourigenesis 78, 79.

Filopodia can also support invasive migration of cancer cells; the local co-trafficking of α5β1 and receptor tyrosine kinases (RTKs, including epidermal growth factor receptor 1) facilitates crosstalk between adhesion receptors and RTKs [80] and supresses Rac activity, but activates RhoA at the leading edge to generate actin-spike protrusions at the front of invading carcinoma cells [81]. Actin-spike protrusions are also formed in response to RhoA activation in breast and lung carcinoma cell lines which express gain-of-function mutant p53, and are clearly distinct from lamellipodia, lacking dendritic actin veils and consisting of numerous short filopodia emanating in the direction of migration in cells moving in 3D-ECMs and *in vivo* [63]. Filopodial actin spikes require the formin FHOD3, which is activated by phosphorylation downstream of RhoA–ROCK, and the density and organisation of filopodia within these protrusions could suggest that they play a role in generating protrusive force.

### Actin Regulators in ECM Remodelling

The ECM acts as a physical barrier to cells, whether presented as a basement membrane surrounding tissues or as fibrillar collagen-based interstitial matrix [82], and although leukocytes (and amoeboid cancer cells which use hydrostatic pressure and membrane blebs to move) appear to move through the ECM in a protease-independent fashion, mesenchymal cancer cells must clear their path by focalising degradative activity. MT1-MMP is a membrane-anchored matrix metalloprotease that plays a particularly significant, nonredundant role in the invasion of a range of cancer cell types [83], and while the leading protrusion of invasive cancer cells may have the capacity to recruit and align ECM fibres (without large-scale degradation), an integrin and actin-rich zone of collagen degradation posterior to this (in front of the nucleus) has been described [84].

A prominent role for the Arp2/3 activator N-WASP in focal proteolysis has been described; N-WASP mediated actin polymerisation promotes the recruitment of MT1-MMP to ‘actin hotspots’, accumulations of F-actin at sites of ECM contact. MT1-MMP is tethered to these actin hotspot foci through an actin-binding domain within the cytoplasmic tail, and thus N-WASP mediated actin polymerisation directs protease activity by generating actin hotspots in close proximity to matrix fibrils destined for degradation in invasive cells [85]. Interestingly, the WASP family member WASH promotes Arp2/3-mediated actin polymerisation on late endosomes, and generates tubules that fuse with the plasma membrane at sites of cell matrix adhesion [26]. Thus, distinct Arp2/3 nucleation promoting factors, acting at different subcellular locations, might coordinate a matrix degradation programme at sites of ECM contact to remove the ECM barrier and facilitate protrusion. Given that **matrix pore size** is a major constraint to translocation of migrating cells [86], it is interesting to speculate that sites of cell–matrix contact in front of the nucleus may act as a constriction band released by such focal proteolysis mechanisms in invasive cancer cells.

## Moving the Nucleus in 3D Matrix

Translocation of the nucleus is often the measure by which cell biologists determine the repositioning of migrating cells, and the nucleus shows a characteristic rearward movement in fibroblasts as they polarise in the direction of migration [87], suggesting that direct mechanisms exist to move the nucleus in migrating cells. Disrupting the LINC (linkers of the nucleoskeleton to the cytoskeleton) complex between the nuclear envelope and cytoskeleton alters microtubule organising centre (MTOC) positioning [88] and inhibits the polarity of fibroblasts [89]. Reorientation of the nucleus in fibroblasts is considered to precede Golgi reorientation [90], an important indicator of polarity in migrating cells. Moreover, in cells migrating within confined spaces (mimicking matrix pores), the nucleus is squeezed and can rupture, suggesting that forces are exerted directly on the nucleus 91, 92.

### Bringing up the Rear: Force Coupling and the Nucleus

In order to enable cell movement in 3D, intracellular organelles have to morphologically adapt. The role of cytoplasm-spanning organelles like endoplasmic reticulum (ER) and mitochondria is largely unexplored, although plasma membrane–ER contact sites have been described to respond to matrix interactions and cell migration [93], and mitochondria seem to preferentially localize to protrusions where energy demand is increased [94]. The biggest obstacle to effective 3D migration, however, is the nucleus. The nucleus is subject to direct actomyosin-mediated forces [95], confirming the central connective role for the nucleus [96] within cytoskeletal rearrangements predicted by modelling and that actin has an active role in transmitting force directly to the nucleus.

In a landmark study, Wolf *et al*. showed that cell movement in a 3D matrix is limited by pore size due to the restrictive dimensions of the nucleus [86]. Specialised cells like neutrophils and dendritic cells have flexible nuclei that are capable of deforming into thin cables through their adaptable lamin networks [86] and perinuclear actin accumulation [97], allowing them to move through small pores in the ECM. However, nuclei of invading carcinoma cells have different mechanical properties due to the composition of their nuclear lamina, especially lamin A/C, and deform to a lesser extent. In a matrix with pore cross sections below ∼7 μm^2^, cancer cells must digest matrices with proteinases to move [86].

Nuclear shape and structure vary greatly – within its spherical constrains – between tissues and is often used in pathological tissue assessments [98]. The viscosity of the nucleus differs from the surrounding cytoplasm 99, 100, and interphase nuclei respond in several ways to migration and the ECM. Inside the nuclear envelope, a network of short lamin filaments 101, 102 supports the membrane and has a direct protein–protein interaction network to the cytoplasm via the LINC complexes, consisting of KASH, like Nesprin 1–4, and SUN domain family proteins (reviewed in 103, 104, 105, 106).

The nucleus itself and perinuclear actin respond to compressive force [107] and this can lead to changes in gene expression. Nuclear lamin expression can adapt to the stiffness of the ECM [108] and chromatin is attached to nuclear lamins [109] and thus has a potential connection to the cytoskeleton and with it changes in force applied to the nucleus. In addition, transcription is sensitive to the stiffness of the environment; specialised transcription factors like TWIST, YAP/TAZ, and SRF react to changes in the actomyosin cytoskeleton and mechanical forces translated from the ECM 110, 111, 112 and factors influencing actin dynamics (e.g., Zyxin and Rac) are mechanosensitive and can play roles in the nucleus 113, 114. Thus, the nucleus may act as a brake on cells migrating in 3D, but physical stimuli can influence nuclear mechanics and gene expression to promote cell movement.

### Cell Motility, Polarity, and the Nucleus in the ECM

Disruption of the nucleocytoskeletal linkage results in impaired migration in restrictive 3D environments, indicating that movement of the nucleus is an active process 89, 115. The force applied to the nuclear membrane has to be able to move the nucleus in the direction of migration: the nucleus could be pushed, pulled or – in a 3D environment – moved along like on a conveyor belt through connections or friction with the plasma-membrane-associated cytoskeleton. Observations of lymphocytes suggest an accumulation of actin behind the nucleus in these cells, which is required for forward pushing of the nucleus, although direct force measurements are lacking [31]. By contrast, experiments with migrating fibroblasts in a nonrestrictive 2D environment, which were unable to detach their trailing edge, were still able to move the nucleus forward, indicating that such nuclei were – at least partially – pulled forward by actomyosin [116].

The emergence of *de novo* actin networks around the nucleus when cells squeeze through tunnels, or during squashing of cells, suggest that the nuclear envelope has an active role in responding to mechanical stimuli 97, 107 and that friction with the cellular surroundings can influence nuclear movement.

### Regulation of Nuclear Dynamics by Actin Regulators

TAN lines are stress fibres crossing the nuclear envelope as part of a perinuclear actin cap that is also present in cells in 3D cultures 115, 117, 118. Actin regulators associated with the nuclear envelope are able to change the characteristics of existing actin filaments to support nuclear movement and force transduction. The Rac GEF STEF/TIAM2 localises to the nuclear envelope, and controls perinuclear Rac activity to regulate actin dynamics and contractility at this subcellular region [119]. Furthermore, the actin-bundling activity of FHOD1 [120] and fascin [121] can support the formation of thick actin fibres associated with the nucleus. FHOD1 is a member of the diaphanous-related formins, but no actin polymerisation ability has been observed to date; by contrast, mDia2, another member of the formin family, is also able to associate with the nuclear envelope and polymerise actin [122]. In elegant experiments using a bead attached to an **AFM cantilever** to push the cell in a directional manner, INF2 (inverted formin 2) was shown to induce a perinuclear actin network that was not only prominent on the nuclear envelope but also extended to regions of ER accumulation and is dependent on Ca^2+^ but not on classical mechanostimuli like non-muscle myosin IIA [107]. Non-muscle myosin IIB activity, by contrast, is required for physical translocation of the nucleus 123, 124 and the unconventional myosin 18A associates with stress fibres stretching across the nucleus [125], suggesting active regulation of actomyosin contractility from the nucleus. Additional actin regulators, like IQGAP1, have been described on the cytoplasmic face of the nuclear envelope without describing a potential function yet [126].

## Concluding Remarks and Future Perspectives

It is clear that while great strides have been made in our understanding of the multifaceted roles of actin in cell migration in 3D, there are still many open questions (see Outstanding Questions). In particular, the nuances of isoform specificity (non-muscle myosin II, actin, and Arp2/3) and emergent properties arising from macromolecular cytoskeletal organisation have not been investigated in 3D migration. Many issues will potentially be answered in the near future through advances in imaging at high spatial and temporal resolution in complex 3D environments and *in vivo*, including lattice light-sheet and super-resolution techniques. It is crucial that the context of 2D migration is better understood in 3D; for instance, the force bearing properties of bundled collagen fibres and basement membranes as migratory surfaces are not well appreciated, and whether these are fundamentally linked to specific types of cell–matrix adhesion (e.g., integrin versus non-integrin) is not clear. Our more detailed knowledge of actin polymerisation networks now makes it possible to infer the dynamic responses of cells to challenge, through forces and/or changes in the topology of the environment. Furthermore, the emerging central role of the nucleus adds a further dimension to the regulation of motility in physiological environments by actin structures. Understanding the mechanisms that govern cell migration in 3D matrices will provide insight into this crucial aspect of development. Manipulating cell migration may also prove useful in regenerative medicine, by targeting stem cells to specific niches (and arresting them there), but also in generation of antimetastatic therapies, which is of paramount importance because metastatic dissemination is the leading cause of death in 90% of cancer patients [127].

Outstanding Questions How do different isoforms of actin, non-muscle myosin II, and the Arp2/3 complex impact on migration in 3D matrices?

Is the ‘clutch’ model of retrograde flow engagement and traction force generation conserved in 3D migration?

Do filopodia in 3D matrices bear or generate force?

Is force exerted on the nucleus in cells moving in 3D matrices?

## Glossary

**2D substrate**: a flat 2D surface (e.g., glass or plastic cell culture vessel) upon which cells can move.
**3D matrix**: a 3D hydrogel or tissue environment.
**AFM cantilever**: probe used to measure physical properties in atomic force microscopy.
**Amoeboid**: mode of migration most often seen in 3D matrices, whereby actomyosin contractility increases hydrostatic pressure to generate membrane blebs.
**Arp2/3**: heptameric protein complex that polymerises new actin filaments as branches from existing filaments, activated by WASP family members.
**Basement membranes**: thin, dense layer of matrix that supports lines surfaces, supporting epithelia, epidermal, and endothelial cells and actin as a boundary within and between tissues. Rich in laminins and collagen IV.
**Blebbing**: spherical expansions of plasma membrane devoid of actin filaments, caused by increases hydrostatic pressure and cytoplasmic flow. Found in apoptotic cells, and used as a means of protrusion/force generation in amoeboid migration.
**Ena/VASP**: family of proteins (including Mena) that associate with barbed ends of actin filaments and prevent capping, promoting F-actin elongation.
**Epithelial–mesenchymal transition**: developmental and tumourigenic programme through which epithelial cells acquire mesenchymal traits, including motility.
**Fascin**: actin-bundling protein that promotes filopodia formation and cancer cell invasion and metastasis.
**Filopodia**: needle-like protrusions made by bundling of F-actin filaments.
**Focal adhesion**: plaque-like structures through which the cytoskeleton links to the ECM via integrins and associated proteins.
**Formins**: family of actin-polymerising and/or -bundling proteins, some of which act as direct RhoGTPase effectors.
**Haptotaxis**: migration within a gradient of matrix ligand.
**Interstitial matrix**: ECM found in supportive and connective tissue, usually rich in fibrillar collagens. Can range in density and rigidity (e.g., tendon and dermis).
**Invadopodia**: actin-rich protrusions that direct matrix degradation, mostly clearly observed beneath cells and perhaps contributing to basement membrane degradation.
**Lamellipodia**: fan- or wave-like protrusions assembled by Arp2/3-mediated actin polymerisation into branched networks.
**Lamellipodin**: Ena/VASP ligand that also interacts with the WAVE regulatory complex to coordinate Arp2/3 activity. Localises to the leading edge of lamellipodia.
**Matrix pore size**: gaps between collagen fibrils (or within basement membranes), which vary dependent on tissue/matrix density.
**Mesenchymal**: mode of migration characterised by elongated morphology in 2D and 3D, and requirement for proteases in 3D migration and invasion.
**Myosin X**: unconventional myosin that binds to and bundles actin filaments. Plays a role in filopodia formation, found at the tips of filopodia.
**Rho GTPases**: family of GTPases considered to be master regulators of the cytoskeleton.
**WASP**: family of Arp2/3-activating proteins that are often effectors for RhoGTPases.
